# Dynamics of patents, orphan drug designation, licensing, and revenues from drugs for rare diseases: The market expansion of eculizumab

**DOI:** 10.1371/journal.pone.0247853

**Published:** 2021-03-05

**Authors:** Rosângela Caetano, Marilena Cordeiro Dias Villela Correa, Pedro Villardi, Paulo Henrique Almeida Rodrigues, Claudia Garcia Serpa Osorio-de-Castro

**Affiliations:** 1 Departamento de Política, Planejamento e Administração em Saúde, Instituto de Medicina Social, Universidade do Estado do Rio de Janeiro, Rio de Janeiro, Brazil; 2 Departamento de Políticas e Instituições de Saúde, Instituto de Medicina Social, Universidade do Estado do Rio de Janeiro, Rio de Janeiro, Brazil; 3 Departamento de Política de Medicamentos e Assistência Farmacêutica, Escola Nacional de Saúde Pública Sergio Arouca, Fundação Oswaldo Cruz, Rio de Janeiro, Brazil; University of Almeria, SPAIN

## Abstract

**Background:**

This study examines the dynamics of the eculizumab patenting, orphan designation, and marketing authorization process in different countries and regulatory systems and analyzes drug revenues since its first marketing authorization.

**Methods:**

A retrospective case study was conducted. Multiple information sources were used to: determine the status of eculizumab patents; examine the designation of orphan drug status by US, European, Japanese, and Brazilian regulatory authorities to determine registration status and approved clinical indications; estimate the prevalence of associated clinical conditions; investigate the history of the drug manufacturer, Alexion Pharmaceuticals, Inc., and its financialized business model; and examine global eculizumab sales revenues since its first marketing authorization.

**Results:**

Our search yielded 32 patent families divided into 98 applications. The first patent granted was filed in 1995 by Alexion Pharmaceuticals, Inc. in the US. Eculizumab has always been as an orphan drug, except in the Brazilian regulatory agency. All clinical indications approved thus far refer to rare diseases (e.g., paroxysmal nocturnal hemoglobinuria syndrome, atypical hemolytic-uremic syndrome, refractory and generalized myasthenia gravis, and neuromyelitis optica spectrum disorder). Alexion’s revenues amounted to more than US$25 billion between 2007 and 2019, showing a growing trend. Eculizumab led sales from the beginning, being the only product in the company’s portfolio until 2015. In 2019, the drug accounted for 79.1% of all revenues.

**Discussion:**

Our findings show that a strategy focused on obtaining orphan drug designation, expanding therapeutic indications and the geographic range of marketing approvals, extending monopoly periods, and prioritizing public procurement niches has enhanced revenues and helped the company achieve leadership in a highly specific and profitable market.

## Introduction

This case study focuses on eculizumab and the regulatory and financial aspects of the drug’s development trajectory. Eculizumab is a drug approved to treat rare diseases, and many aspects related to its patenting, marketing authorization, therapeutic indications, and pricing render the study of this medicine relevant as an exemplary case concerning orphan drugs. The clinical indications for eculizumab have progressively expanded over time and share a common characteristic–rare diseases. Since its first patent in the US the number of patent applications filed in intellectual property offices in other countries has multiplied. Eculizumab has been described as the most expensive drug in the world, with an estimated cost of US$ 410,000 per patient/year in the US in 2010 [1]. The development of the molecule and drug marketing strategy were guided by a financialized business model [2–4] adopted by the patent owner, Alexion Pharmaceuticals, Inc, in 1992.

Although rare diseases are usually associated with relatively low incidence and prevalence, definitions vary by country or region [5, 6]. In the European Union (EU), a disease is considered rare when it affects no more than 5 in 10,000 people, while in the US the term rare disease means any disease or condition that affects less than 200,000 people (or 7.5 in 10,000 people). Diseases with an even lower prevalence (less than 10 cases per million) are termed ultra-rare diseases [7].

Rare diseases may be considered unimportant from an epidemiological perspective. However, when the individual prevalence rates of all rare conditions (there are between 5,000 and 8,000 rare diseases) are added together, the number of patients living with these diseases is significant. Experts estimate that the sum of the prevalence of all rare diseases is approximately 5 to 8%, meaning that over 400 million people worldwide are affected by these conditions [8].

Drugs developed to treat rare diseases are called “orphan drugs” [9]. Owing to the low frequency of these diseases, sponsors are generally reluctant to develop these medicinal products under usual patenting, marketing authorization, and commercialization conditions. The underlying assumption is that these drugs provide low economic return on the investment in research and development (R&D) made by the pharmaceutical company/patent holder.

Several jurisdictions (e.g., the US, EU, Japan) have developed specific policies and legislation to encourage the development of orphan drugs [10]. Table 1 provides further information on orphan drug definitions, additional criteria for assignment of orphan status, exclusivity and registration, and economic incentives for the development of orphan drugs in these jurisdictions.

**Table 1:** Selected aspects related to rare diseases and orphan drugs according to jurisdiction.

Selected aspects	US	EU	Japan	Brazil
Specific orphan drug designation	Yes	Yes	Yes	Yes
Definition of rare disease based on patient populations	<200,000 patients^†^	<5 in 10,000 individuals^‡^	<50,000 patients^¥^	<65 patients/100,000 individuals^§^
Additional definition criteria	i) Disease or condition that affects >200,000 people for which there is non´t any reasonable expectation that the US development and distribution costs are will be recovered by from sales (ii) Vaccines, diagnostics or specific drugs for the prevention of a rare disease or condition^†^	(i) Drug intended for life-threatening, seriously debilitating or serious and chronic conditions that without incentives it is unlikely that the marketing of the product would generate sufficient return to justify the necessary investment (ii) Drug intended for the diagnosis, prevention, or treatment of a rare disease in the absence of other methods of diagnosis, prevention, or treatment, or if the drug is safer, more effective, or clinically superior^‡^	(i) High probability of development, supported by theoretical justification and realistic development schedule (interest of the company in developing the hypothetical orphan drug) (ii) No alternative drugs or treatments for the diseases, or need for greater effectiveness and/or safety^¥^	Drugs intended for the treatment, diagnosis or prevention of rare diseases used for serious debilitating conditions and that significantly alter the clinical evolution of the disease or enable remission^§^
Orphan drug exclusivity	7-year market exclusivity from date of approval	10-year market exclusivity from date of approval	10-year registration validity period and 10-year exclusivity period for collected data	No exclusivity
Economic Incentives (reduced R&D costs, tax credits, and fees)	(i) 50% tax credit on R&D costs; R&D grants for Phase I to Phase III clinical trials. (ii) User fee waivers (for companies with an annual revenue of < $50 million)	(i) Reduced fees for EMA protocol assistance. (ii) Reduced fees for administrative and procedural assistance for small and medium-sized enterprises. (iii) Does not offer research grants^θ^.	(i) Up to 50% of orphan drug development costs subsidized^α^. (ii) PMDA charges reduced user fees for guidance and consultations. (iii) 12% of study expenses (not including grant subsidies) can be reported as a tax credit.	No incentives
Alternative procedures for orphan drug registration	Fast-track, accelerated approval and breakthrough therapy	Conditional approval, adaptive pathways	Accelerated review procedure for new drug approval applications	ANVISA expedited procedure with regulatory flexibilities (proof of safety and efficacy) and conditional approval^§^

Sources: Adapted from Minghetti, Giudici and Montanari [11], and EvaluatePharma [12].

US, United States of America; EU, European Union; R&D, Research and development; EMA, European Medicines Agency; PMDA, Japanese Pharmaceuticals and Medical Devices Agency; ANVISA, Brazilian Health Surveillance Agency.

^†^ Orphan Drug Act 1983 [13]

^‡^ European Community Regulation No. 141/2000 [14]

^θ^ The EMA does not offer research grants but funding is available from the European Commission and other sources (Horizon 2020 and E-Rare, for example)

^¥^ Orphan Drug Act 1993 [15, 16]

^α^ Up to 50% of orphan product development costs may be subsidized in Japan through the National Institute of Biomedical Innovation

^§^ ANVISA RDC No. 205/2017 [17].

Orphan medicinal product designation precedes regulatory approval. Pharmaceutical companies developing health technologies for rare diseases request designation because this status makes them eligible for incentives and benefits to develop and commercialize these products [18].

Eculizumab is a humanized monoclonal antibody that binds with high affinity and specificity to the terminal complement protein C5, consequently blocking the inflammatory response and impacting the body´s ability to attack and destroy blood and kidney cells [19]. The first studies on this drug were conducted by Leonard Bell, professor in the Department of Pathology and co-Director of the Vascular Biology Program at the Yale School of Medicine [20], and Steven Squinto, professor of biochemistry and molecular biology at Louisiana State University Medical Center [21]. The development of eculizumab was completed by Alexion Pharmaceuticals, Inc, co-founded by Bell and Squinto, who remained with the company until 2015. Since its development, eculizumab has obtained orphan designation for different indications in various jurisdictions.

Complement-specific therapy has opened up a huge novel drug market. Because the range of complement-related therapy is so broad, potential indications for complement inhibition identified by use of off-label indications of drugs already on the market are expanding [22–24]. Other promising indications are also undergoing testing and new indications are in the pipeline. However, eculizumab has been the primary revenue generator among the drugs of its class in the past ten years [25].

Orphan drugs are typically sold at relatively high prices [26]. However, how pharmaceutical companies set these prices remains a mystery. Substantial revenues obtained by manufacturers have been mentioned elsewhere in the orphan drug literature [25, 27]. Regarding eculizumab, a 2016 report estimated that total annual expenditure for a typical patient with paroxysmal nocturnal hemoglobinuria based on estimates of annual utilization calculated using the standard FDA-approved dosing ranged from US$432,240 to US$542,640 per patient [28].

This paper highlights specific issues discussed in the literature, bringing together comprehensive information on eculizumab and clearly charting the company’s success in disseminating uses to boost revenue. This study examines the dynamics of the eculizumab patenting, orphan designation and marketing authorization process in different jurisdictions and analyses eculizumab sales revenues since its first marketing authorization.

## Method

We conducted a retrospective single-case study. Case studies are useful for describing complex social issues. They enable the researcher to explore real-life events, making it easier to understand the dynamics of specific contexts and allowing for an in-depth description of the events or phenomena in question [29].

Multiple information sources were used to perform the following steps: (i) a search for eculizumab patents and their status at several international patent offices, (ii) an assessment of the orphan drug designation process in the main international regulatory authorities, (iii) an assessment of marketing authorization status and clinical indications approved by these agencies, (iv) estimation of the prevalence of associated clinical conditions, (v) brief description of the history of Alexion Pharmaceuticals, Inc. and its business model, and (vi) analysis of the company’s global eculizumab sales revenues since its first marketing authorization.

An initial search for eculizumab patent applications was carried out between December 2019 and January 2020 using the Integrity Database [30], which lists applications according to international nonproprietary names (INN). The name of the active substance (i.e., eculizumab) was used in the search. The search results were the WO (short for WIPO) patent application numbers assigned by the World Intellectual Property Organization (WIPO) after the filing of an international patent application under the Patent Cooperation Treaty (PCT). This treaty allows the applicant to file simultaneous patent applications in the cosignatory countries. We then conducted a search for patent families, which are collections of all patent applications covering the same invention. Some patent families were related to applications originally filed in the US.

The patents corresponding to the WO application numbers were then obtained from the databases of the following international patent offices: the United States Patent and Trademark Office (US) [31], the European Patent Office (Espacenet) [32], the Japan Patent Office (Japan) [33], and Brazil’s National Institute of Industrial Property [34]. Google Patents [35], which indexes patents and patent applications in several patent offices, including WIPO and the European, Japanese, Chinese, and Canadian offices, was also used, allowing for cross-checking of data to improve accuracy.

The study was restricted to patents filed by Alexion Pharmaceuticals, Inc., the patent holder and manufacturer of eculizumab. All duplicate patents were excluded. We recorded the filing dates and patent/patent application status. The latter was classified as active, pending, or abandoned, based on the information found on each database.

Orphan drug status was verified at the US Food and Drug Administration (FDA) [36], the European Medicines Agency (EMA) [37], the Japanese Pharmaceuticals and Medical Devices Agency (PMDA) [38], and the Brazilian National Health Surveillance Agency (ANVISA—*Agência Nacional de Vigilância Sanitária*), based on each recorded clinical indication.

A search for marketing authorization dates and approved clinical indications for eculizumab was conducted on the US [39], European [40], Japanese [41], and Brazilian [42] agencies’ websites using the drug name as the keyword.

The market potential of the drug is related to the prevalence of the rare diseases for which eculizumab has an approved clinical indication. Data on prevalence per 100,000 population was obtained from the websites of two rare disease initiatives: (i) Orphanet, a network established in France in 1997 and subsequently expanded by grants from the European Commission to a consortium of 40 countries in Europe and worldwide [43]; (ii) the National Organization for Rare Diseases, that articulates advocacy organizations for patients with rare diseases, connects doctors and patients, funds treatments and consultations, and engages in advocacy with US policymakers [44]. We opted to use different sources of information for two reasons: first, the prevalence of certain rare diseases is not well known; second, disease prevalence varies across regions and large discrepancies exist between the sources.

Data on Alexion Pharmaceuticals, Inc. were obtained from the company’s website [45], NASDAQ website [46] and available literature on business models [47, 48] and the financialization of the US pharmaceutical industry [3, 4], focusing on pharmaceutical companies that develop biologic agents [49]. Data on total revenue and revenue by pharmaceutical product were taken from the company’s annual financial reports (from 2007, the year that eculizumab was first launched, to 2019) obtained from its website in two tabs: “News & Media” [50] and “Financials” [51]. Values were extracted in millions of US dollars and the revenue attributed to eculizumab was calculated relative to total annual revenue. In addition, product market expansion was analyzed by comparing eculizumab sales revenue with the annual number of clinical indications approved by international regulatory agencies.

Data on eculizumab procurement by federal government bodies in Brazil between January 2007 and December 2019 were extracted from the records of the Integrated General Services Administration System (*Sistema Integrado de Administração de Serviços Gerais–*SIASG). SIASG contains detailed information on all items purchased by the federal government, including supplies and services [52]. We obtained data on the number of purchases, quantities purchased, and annual purchase values. All values were converted to US dollars using the annual average exchange rate (Brazilian real [BRL] per US dollar [US$]) published by the Central Bank of Brazil obtained from the Institute for Applied Economic Research (*Instituto de Pesquisa Econômica Aplicada*) [53].

Finally, eculizumab sales revenues worldwide and in Brazil were examined to highlight the importance of the Brazilian market to overall eculizumab sales revenues.

Only publicly available data were used in this study.

## Results

Eculizumab’s patent profile included a total of 32 patent families divided into 98 applications. The first patent granted was filed in the US in 1995, with Alexion Pharmaceuticals, Inc. as the depositor and patent holder.

**Fig 1:**
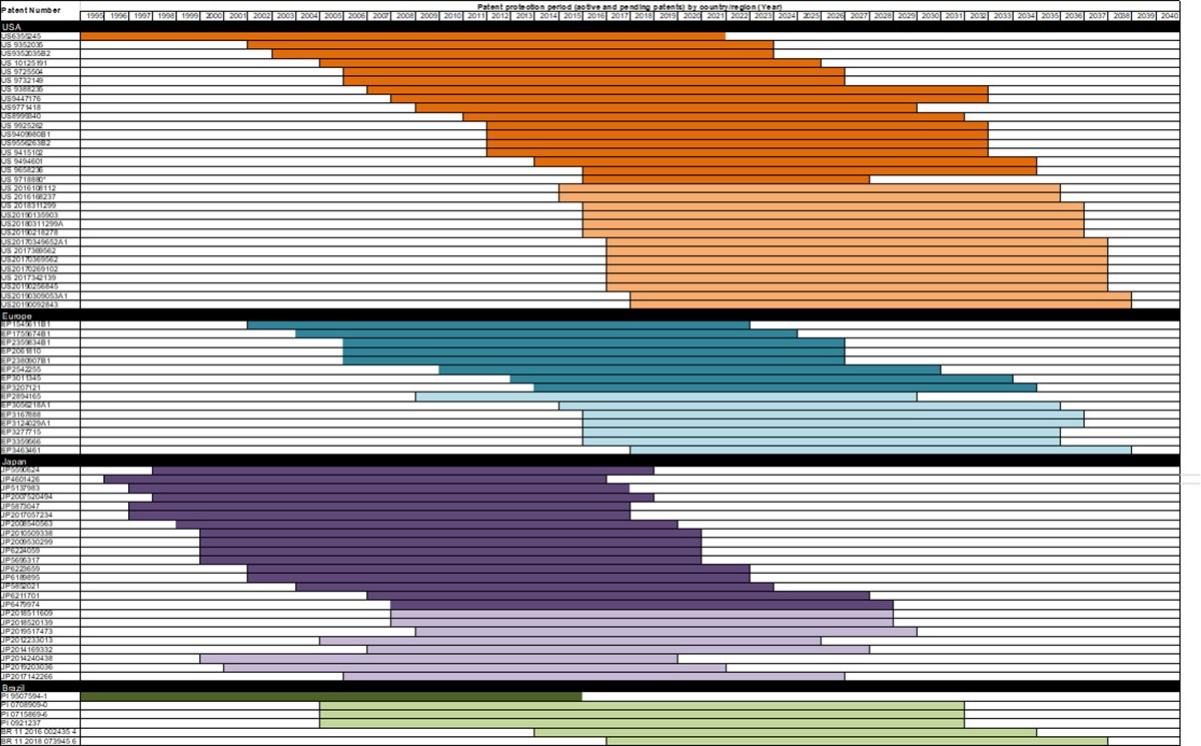
Active and pending eculizumab patents deposited at patent and trademark offices in the United States, European Union, Japan, and Brazil up to 2019. Note: Fig 1 provides an overall view of the duration of protection granted to each active patent in each jurisdiction (illustrated by darker horizontal lines) and the estimated duration of pending patents (illustrated by lighter horizontal lines). The patent numbers are displayed in the right column and the duration of protection (in year) is shown at the top.

The US has 17 active patents from 1995 to 2034. The EU has eight active patents, which guarantee exclusivity from 2003 to 2035. Japan has 16 active patents from 2005 to 2037 (Fig 1).

In the case of pending patents—applications still under examination at the respective patent office for various reasons (e.g., requirements, litigation, and examination delays)—monopoly periods may be extended if the patent is subsequently granted. The monopoly period for subsequently granted patents may be extended to 2039 in the US (14 pending patents), 2038 in the EU (7 pending patents), and 2029 in Japan (8 pending patents). This means that Alexion’s monopoly on eculizumab will be extended for up to 44 years if all pending patents are subsequently granted (Fig 1).

In Brazil, the first patent application was filed in May 1995 and granted in August 2010 (expiration date May 2015). Six other patent applications were found in Brazil, filed between 2006 and 2018. None had been granted by the date of completion of this study. Three of these applications fall within the sole paragraph of Article 40 of Brazil’s Industrial Property Law (IPL). The law provides that a patent shall be valid for at least 10 years from the grant date [54], regardless of the application filing date, meaning that eculizumab could have monopoly status in Brazil for 32 years.

**Fig 2:**
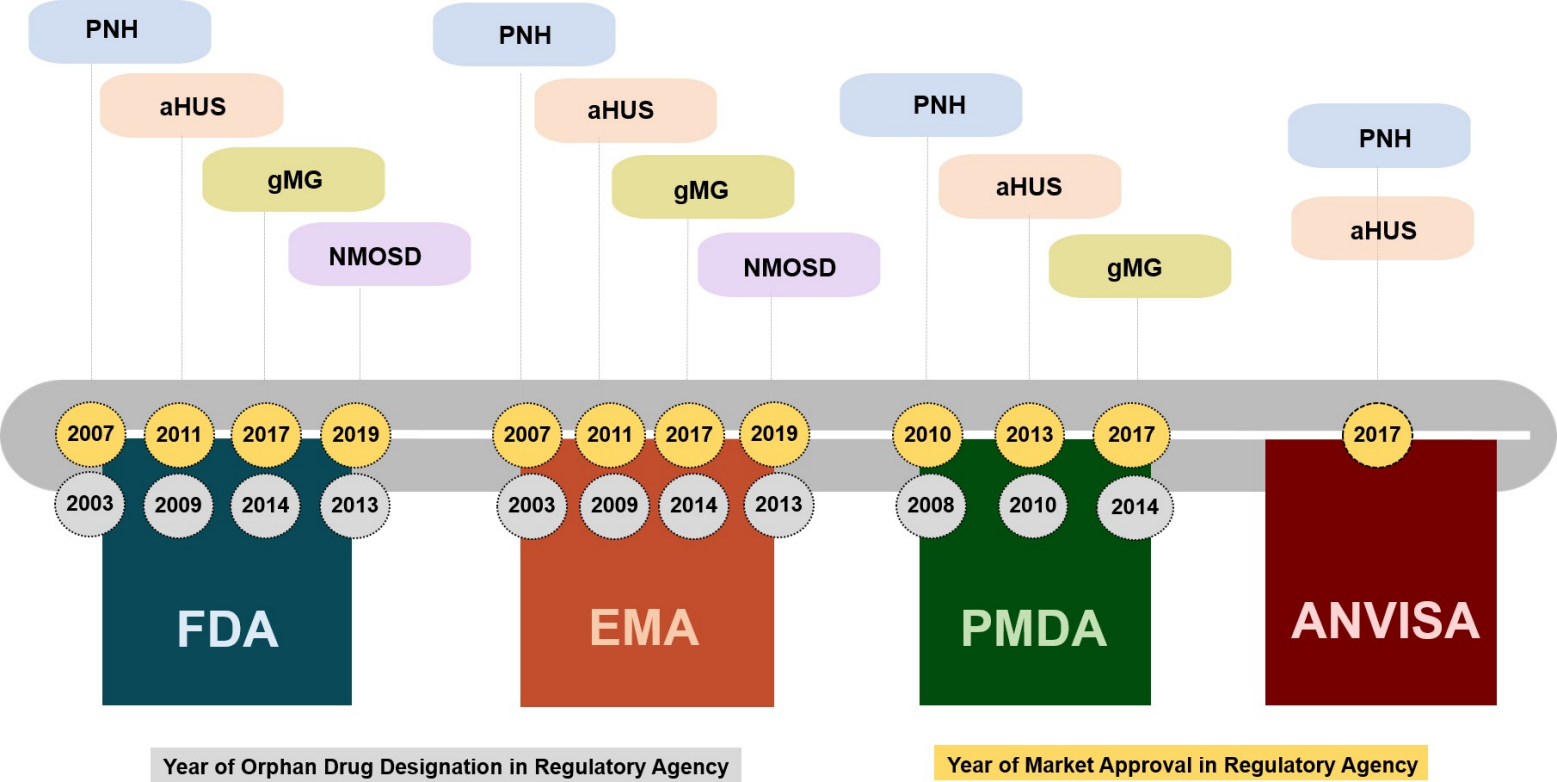
Timeline of eculizumab orphan drug designation and marketing authorization in international regulatory agencies according to year and indication, 2007–2019. Paroxysmal Nocturnal Hemoglobinuria (PNH), Atypical Hemolytic Uremic Syndrome (aHUS), Generalized Myasthenia Gravis (gMG), Neuromyelitis Optica Spectrum Disorder (NMOSD), FDA–Food and Drug Administration; EMA–European Medicines Agency; PMDA—Pharmaceutical and Medical Devices Agency; ANVISA–Brazilian Health Surveillance Agency.

As of January 2020, eculizumab was registered for four clinical indications: paroxysmal nocturnal hemoglobinuria (PNH), atypical hemolytic-uremic syndrome (aHUS), generalized myasthenia gravis (gMG), and neuromyelitis optica spectrum disorder (NMOSD) (Fig 2).

Approved by the FDA and EMA in 2007 with only a three-month interval between approvals, PNH was the first clinical indication for eculizumab. Since then, the number of clinical indications approved by these and other regulatory agencies worldwide has expanded. Eculizumab was approved in the US and EU for the treatment of NMOSD only in mid-2019, and is still under evaluation by Japan’s PMDA. It is worth highlighting the long period of time (ten years) between the granting of the first marketing authorization for eculizumab by international agencies (2007) and the granting of authorization by the Brazilian agency ANVISA (2017). The latter is restricted to the two oldest clinical indications, PNH and aHUS (Fig 2).

Brazil had no specific regulations for the licensing of drugs for rare diseases when eculizumab was undergoing the marketing authorization process. In the other agencies that were studied, the drug was always examined considering the orphan designation and prerogatives. The interval between designation as an orphan drug and the granting of marketing authorization ranged from two to six years and was slightly shorter in the PMDA than in the other agencies (Fig 2).

All approved clinical indications for eculizumab to date are diseases classified as rare and, in the case of aHUS, ultra-rare, according to the prevalence estimates shown in Table 2.

**Table 2:** Prevalence of approved indications for eculizumab (per 100,000).

Disease	Orphanet	National Organization for Rare Diseases
Paroxysmal Nocturnal Hemoglobinuria	1-9/100,000	0.05–0.15/100,000
Atypical Hemolytic Uremic Syndrome	0.1–0.9/100,000	0.011/100,000 (Europe, age 0–18 yrs) -0.2/100,000 (US)
Generalized Myasthenia Gravis	1-9/100,000	14-40/100,000 (US)
Neuromyelitis Optica Spectrum Disorders	1-9/100,000	1-10/100,000

US—United States.

Sources: Orphanet— https://www.orpha.net/consor/cgi-bin/Disease_Search.php?lng=PT; National Organization for Rare Diseases— https://rarediseases.org/for-patients-and-families/information-resources/rare-disease-information/.

**Fig 3:**
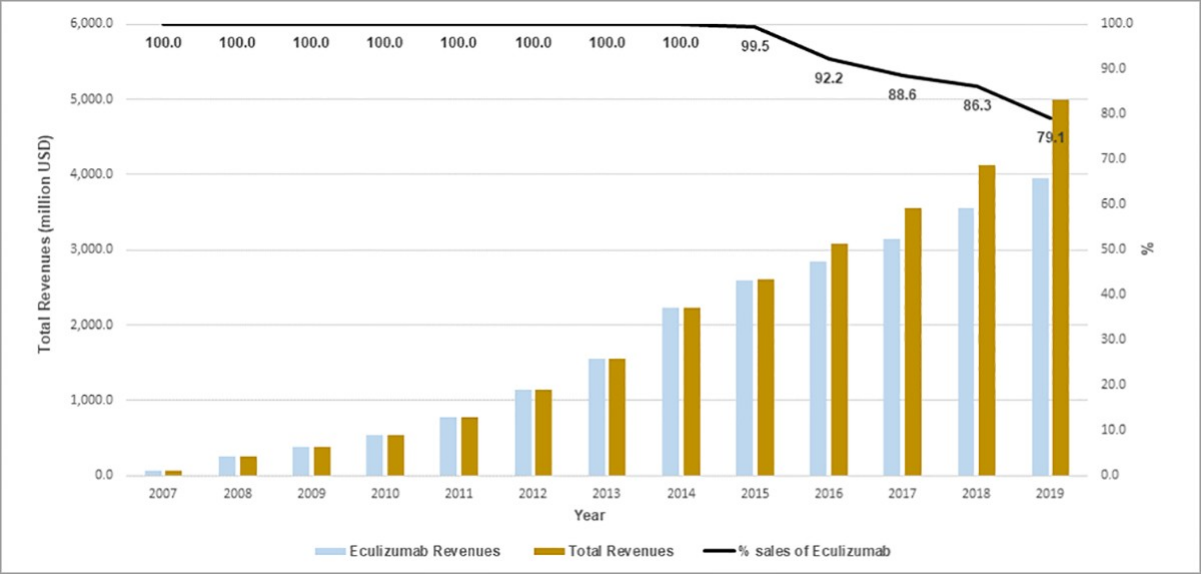
Eculizumab and total revenues of Alexion Pharmaceuticals, Inc. (million US$), 2007–2019.

Alexion’s global revenue has steadily increased since 2007, reaching US$4.99 billion in 2019, with eculizumab sales alone accounting for US$3.94 billion (79.1%) of this amount. Eculizumab was responsible for all the company’s sales revenue until 2014. The proportion of its share in Alexion’s revenue started to decline as of 2015, as the company expanded its pharmaceutical product portfolio (Fig 3 and S1 Table).

**Fig 4:**
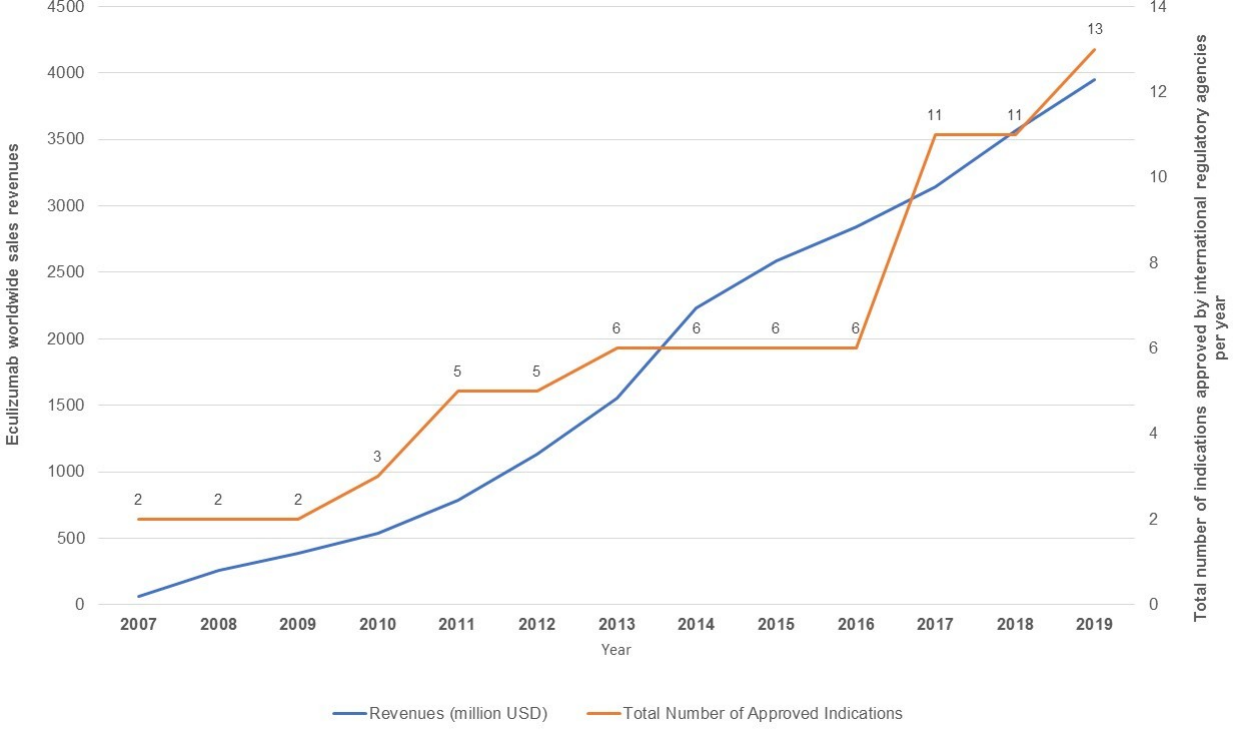
Eculizumab revenues (in million US$) and total number of indications approved by international regulatory agencies, 2007–2019.

The increase in the number of approved clinical indications across different countries influenced eculizumab’s global revenue share, with the results showing a direct relationship between these two factors (Fig 4).

**Fig 5:**
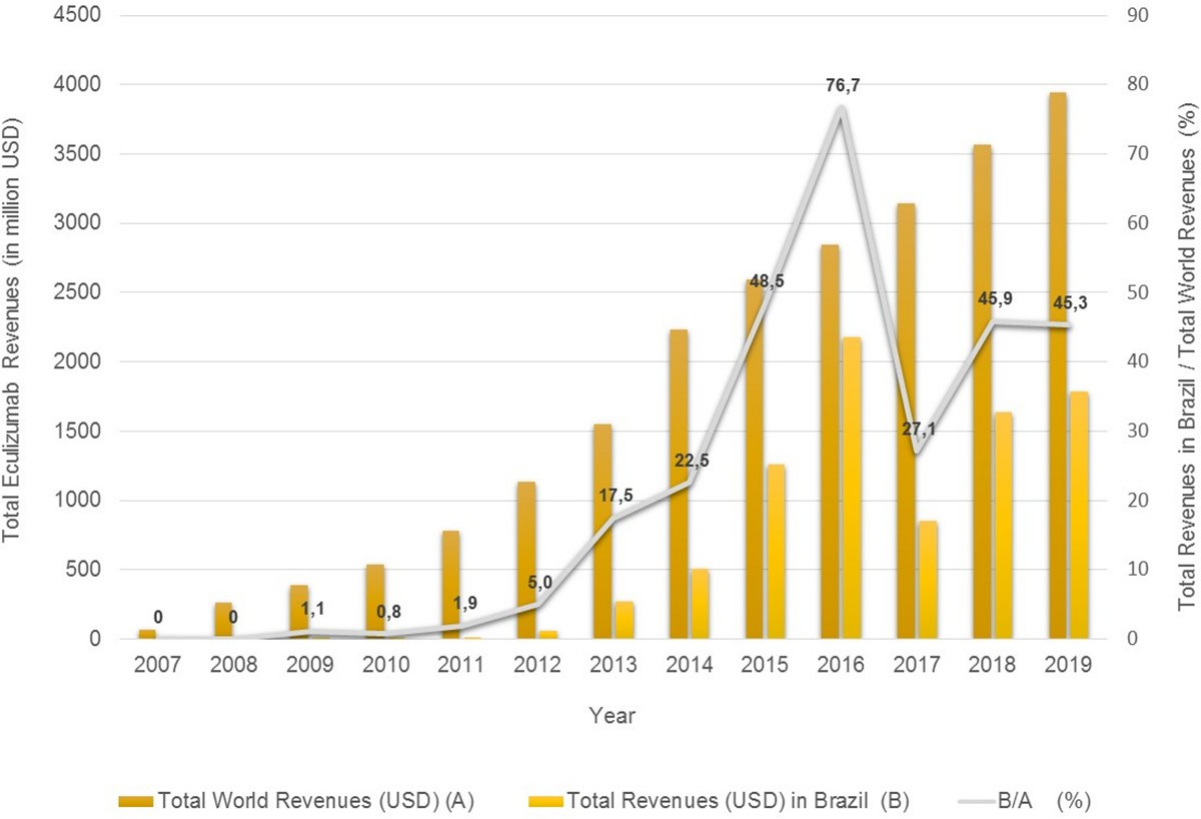
Total eculizumab sales revenue in Brazil and worldwide (in millions of US$). Alexion Pharmaceuticals, Inc., 2007–2019.

Alexion does not publish data on eculizumab sales broken down into number of dosage forms or revenues in different jurisdictions. In Brazil, where due to its remarkably high price the procurement of eculizumab is totally restricted to the public sector, total expenditure on the drug between 2007 and 2019 was US$8,563,184,381.05. These numbers show the important contribution of the Brazilian market to company revenues, representing 76.7% of Alexion’s global revenue in 2016. The share of the company’s global revenue attributable to the Brazilian market only began to drop in 2017, when the drug was granted marketing authorization in the country (Fig 5 and S2 Table).

## Discussion

One of the aims of a business strategy is to secure the extension of patent monopoly. Patent protection, active from the filing date of a patent application, confers exclusive rights over the industrial and commercial utilization of the technology.

Eculizumab’s patent profile and patent status show the extent and duration of the monopoly. The patent data show that the company adopted an aggressive marketing strategy. The results of the patent status search show that the company will have an average of 38 years of marketing exclusivity if all the remaining pending patent applications are approved.

Companies generally adopt two key strategies for extending patents. One is to claim a second (or subsequent) medical use or indication for a known, previously patented chemical compound [55]. The other refers either to small structural modifications to the original molecule or combinations of active or non-active ingredients in a novel pharmaceutical entity. This second strategy is also referred to as “evergreening”, and expands the duration of a monopoly, without improving the drug itself [56]. Both strategies prolong patents and are pursued for commercial ends, impacting generic competition and access to drugs.

The practice of filing several patent applications is common in the pharmaceutical industry and can lead to significant delays in generic availability. Eculizumab’s monopoly periods extend far beyond the 20 years established in the current intellectual property regime—the period within which companies seeks to recover their R&D investments.

Eculizumab is a potential target for the development of biosimilars. As patents on biological medicines expire, non-innovator versions such as biosimilars may enter the market. Due to the threat of litigation between competitors and to secure market exclusivity, companies employ various patenting strategies to extend the patent period and impede new market entries, including second medical use patents. However, second medical use patents are not granted in all jurisdictions. They are permitted in the US and have acquired permission over time in the EU, while in Japan second medical use is patentable material [55, 57]. In Brazil, as a rule, patents for a second medical use are not granted because they are not seen to involve innovation. However, other intellectual property protection mechanisms can also be employed, such as copyright, design rights, trademarks, and trade secrets, creating uncertainty on the launch date of a biosimilar on the market [57].The eculizumab patent expires in 2021 in the US, and soon after in the EU (Fig 1), and at least one biosimilar is currently in development. Amgen has been developing ABP 959 for some time [58], and a phase 3 clinical trial for paroxysmal nocturnal hemoglobinuria is currently underway (ClinicalTrials.gov Identifier: NCT03818607), with an estimated completion date of March 2022. In 2020, Amgen settled a dispute with Alexion over its Soliris biosimilar and is set to market the product in 2025 [59].

In Brazil, the fifteen-year delay between the first patent application and granting the patent prompted Alexion to file a suit to extend the term of patent protection to 20 years from 2010, which was subsequently rejected by the Brazilian courts [60]. The only active eculizumab patent expired in 2015 and only pending patents remain (Fig 1).

Given the large percentage of orphan diseases with unmet needs, one must question whether the current monopoly-based innovation system is the best way to meet the health needs of people affected by these diseases. Other factors may also influence the emergence of biosimilars, such as the complexity of the manufacturing process, differing national regulatory processes, difficulties associated with switching and substitution of biosimilars, local price mechanisms, and volume of expected global sales [61].

Marketing authorization processes in the regulatory agencies allude to a strategy that expands, clinically and geographically, the possibilities of financial gain for the company. In clinical terms, eculizumab was initially registered for a niche indication, with a subgroup of explicitly identified patients. Gradually, new indications involving complement inactivation were added [22–24]. This strategy also expands the market area, with the granting of marketing authorizations in the various international agencies. The strategy is not exclusive to Alexion; several pharmaceutical companies are focusing their efforts on extending the clinical indications for currently available drugs [62].

In most of the regulatory agencies and for all approved indications, marketing authorization requests were filed after eculizumab had received orphan drug designation. In Brazil, however, the law that regulates the licensing of drugs for rare diseases was published only after marketing authorization was granted [63], meaning that the patent holder was unable to request orphan designation.

As shown in Table 1, orphan drug designation provides various benefits to the patent holder, including regulatory and economic incentives. These incentives may be warranted by the rarity of these conditions, making it more difficult for companies to recoup initial R&D costs and discouraging investment.

Incentives vary across jurisdictions and may include periods of marketing exclusivity that differ, distinct from the patent monopoly afforded by the patent [10, 64]. This means that during the exclusivity period regulatory agencies cannot approve generic drugs or other products for the same rare disease; however, this status does not prevent the drug from being approved for a different disease [10, 65].

Some authors consider that this benefit creates negative incentives for the development and marketing of other drugs for the same indication, establishing a market monopoly that allows pharmaceutical companies to charge “exorbitant prices” for orphan drugs [66]. However, there is no consensus on this issue. An in-depth evaluation of the impact of the American Orphan Drug Act from 1985 to 2017 showed that there is little relation between market incentives and drug approvals [67]. Factors such as market size, type of disease, clinical evidence, and the age of onset of symptoms are predictors for the development of other orphan drugs for the same rare indication [68]. In addition, generic manufacturers may believe that orphan drugs fail to provide a large enough return on investment, resulting in a lack of competition and making the additional period of exclusivity for the orphan drug nonessential, even when it exceeds the duration of the patent [67].

On the other hand, increased interest in the development of orphan drugs is driven by several factors [69]. In a study that estimated the differences between trial characteristics and development costs for 100 orphan and 100 non-orphan drugs, Jayasundara found that the costs for orphan products, including new molecular entities, are usually lower [25]. The average cost of developing drugs for rare diseases may be less than half that of developing drugs for other diseases due to flexibility in patent approval, which is based on less rigorous and less costly clinical trials [67]. Orphan drugs show significantly shorter timeline for clinical development and have a greater overall probability of regulatory success (3.9 yrs; 93%) in comparison with non-orphan drugs (5.4 yrs; 88%) [70]. Orphan drug approval success may be related to the previous experience of the drug sponsor, early dialogue with regulators, adherence to recommended changes and pharmaceutical companies’ recognition of the lack of interest from generic manufacturers [67]. Economic incentives and policies seem to have achieved the goal of stimulating orphan drug R&D. The number of drugs for rare diseases has increased in the US and EU [71]. In 2019, 44% of new FDA approvals went to orphan drugs [72].

Despite small markets, companies can dictate high prices for orphan drugs—generally higher than for other undesignated products–since they are the only available drug therapy for rare diseases [73]. The 10 most expensive drugs in the world in 2017 were all orphan drugs for rare diseases and included eculizumab [74]. In 2014 the median differential cost per-patient was 13.8 times higher for orphan drug treatments, and the average annual cost of orphan drugs per patient was $111,820 compared with $23,331 for a non-orphan drugs [75].

All market approved clinical indications for eculizumab correspond to rare and usually severe conditions. PNH is an acquired chronic hemolytic anemia caused by a mutation that leads to the dysregulation of the complement system. Without treatment, between 20 and 50% of patients die within five to six years of diagnosis [76]. Atypical hemolytic uremic syndrome is a debilitating genetic disorder which can lead to systemic complications in several organs. Mortality and morbidity are high in the acute phase, and approximately half the patients develop end-stage renal disease [77]. Myasthenia gravis (MG) is a neurological condition characterized by painless skeletal muscle weakness, fatigue and breathing difficulties, usually caused by antibodies against postsynaptic proteins, including the nicotinic acetylcholine receptor [78]. Eculizumab is only indicated for refractory gMG patients who have tested positive for anti-acetylcholine receptor antibodies, representing a very small segment of patients [79]. NMOSD is an uncommon disease that affects the central nervous system, whereby the immune system attacks the optic nerves and spinal cord. If left untreated, approximately one-third of patients die within 5 years after the first attack [80].

Due to the lack of population-based epidemiological studies on rare diseases, the exact prevalence of clinical conditions with approved indications for eculizumab is not fully known. However, they are all low-frequency conditions. The differences in prevalence shown in Table 2 are possibly caused by the variable clinical manifestations of rare diseases, variations in age, geographic location, and ethnicity, lack of standardized diagnoses, and late diagnosis [81].

Alexion has developed a highly financialized business model [2, 3, 49] based on orphan drugs for rare diseases. This model essentially involves: (i) patenting and marketing authorization in major world markets, (ii) research focused on new clinical indications that generate new patenting processes, (iii) setting prices and defining marketing policies, including those for public health systems, such as in Brazil, and (iv) “predatory value extraction”, which prioritizes distribution of dividends and stock buybacks for shareholders [3, 82]. Alexion is among the top-10 biotechnology companies in the US [4] and allocates significant resources to repurchase its own shares, a key indicator of a company’s level of financialization [83].

The monopoly status and market authorization of eculizumab in relevant markets has turned Alexion into a great commercial success. Its marketing strategy has yielded significant results, with the company’s share price on the NASDAQ rising 600% between 2007 and 2012 [48].

The company began to expand the clinical indications for eculizumab while prospecting for other drugs. Between 2000 and 2020, Alexion acquired seven pharmaceutical companies producing orphan drugs for rare diseases, together with their intellectual property rights, and established partnerships with six others [47, 84, 85]. In 2012, Alexion acquired asfostase alfa, a potential treatment for patients living with HPP (from Synageva), and in 2013, sebelipase alfa, a potential treatment for patients living with LAL-D (from Enobia). In 2015, Alexion obtained approval for sebelipase alfa (Kanuma®), the first treatment for patients with lysosomal acid lipase deficiency (LAL-D), and asfotase alfa (Strensiq®), the first treatment for patients with hypophosphatasia (HPP). As of October 2020, the Alexion pipeline had three products in Phase I trials, five in Phase II and 13 in Phase III [86]. It is reasonable to assume that at least part of retained earnings from products already on the market have been redirected either towards these new commercial ventures or to the expansion of necessary investments in R&D.

Alexion´s total revenue between 2007 and 2019 was more than US$25 billion. Eculizumab has been the company’s flagship product since the beginning and the only product in the company’s portfolio until 2015. After 2016, with increased sales of asfotase alfa and sebelipase alfa, the share of Alexion’s global sales attributed to the drug dropped below 90% and in 2019 eculizumab sales accounted for 79.1% of the company’s revenues. However, these figures do not necessarily reflect the expansion of the drug’s use for NMOSD, an indication approved by the FDA and EMA only in mid-2019.

The development of medicines for rare diseases has become highly profitable. Between 2000 and 2012, orphan drug companies had a 9.6% higher return on investment than non-orphan drug companies [87]. This profitability shows no signs of abating. Orphan drugs revenues account for an important and growing share of the pharmaceutical market, amounting to an estimated US$217 billion in 2024, which corresponds to 18% of prescription sales.

In 2018, Alexion licensed and marketed ravulizumab (Ultomiris®)—a long-acting version of eculizumab with indication for adult patients with PNH—in the US, Japan, EU, Canada, and Brazil. Approval was based on two multicenter phase III clinical trials that established the non-inferiority of ravulizumab to eculizumab [88, 89]. Ravulizumab has the advantage of a longer interval between doses and could potentially replace eculizumab as the first-line treatment for PNH in both treatment-naive and clinically stable patients [90]. However, despite improving patient management, it cannot be considered an actual new drug entry in the field of anti-C5 therapy [23]. Launching ravulizumab may be a strategy to address competitive challenges related to the production of potential eculizumab biosimilars. In November 2020, nine different indications were undergoing testing in Phase 3 trials [86]. Meanwhile, ravulizumab prescriptions exclusively for PNH generated revenues of 814 million dollars (8.9% of the company’s global sales revenues and 10.8% of eculizumab sales revenues) in 2018 and 2019.

This case gains special relevance in Brazil, where public provision of eculizumab is the rule. Alexion took seven years to submit a marketing authorization application, which was finally submitted in 2015). During that time procurement was done exclusively in response to litigation. Marketing authorization was granted in March 2017 [91]. In December 2018, the drug was included on public funding lists, exclusively for the treatment of PNH [92]. The clinical protocol that ensures patient access to the drug under the public health system and regular procurement was published only in November 2019 [93], effectively meaning that procurement up to December 2019 was subject to bidding waiver (152,209 vials purchased by the Ministry of Health for US$8,563,184,381.05) [94].

The decision to delay the submission of the application for marketing authorization may seem paradoxical given the importance of the Brazilian market for company revenues. Before receiving marketing authorization and inclusion on funding lists and clinical protocols, drugs may only be purchased in response to health litigation, an exceptional circumstance that requires a bidding waiver and purchase at market price. Medicine prices are only regulated after the drug receives marketing authorization, when the Drug Market Regulation Chamber (*Câmara de Regulação do Mercado de Medicamentos*–CMED) sets a ceiling price for public procurement. This means eculizumab remained without a price ceiling in Brazil for more than 7 years. After this period, the price was reduced by 35%, resulting in a drop in company revenues.

Delaying the submission of the application for marketing authorization may be regarded as an alternative strategy to extend the exclusivity generated by patent applications. Eculizumab has five patents pending approval, thus inhibiting generic competition [95]. Since there was no dossier to support an application for marketing authorization for a biosimilar, as required by Brazilian law [96], this strategy prevented generic versions of eculizumab from being licensed, generating an additional 11-year exclusivity period that guaranteed the company significant revenues from litigation-related purchases.

This study has some limitations. First, the search was restricted to only four international patent databases, meaning that the information presented may be incomplete. The use of a broader range of databases may therefore have provided a more comprehensive view of the dynamics of patent protection. Second, although data on the prevalence of conditions with approved indications for eculizumab were obtained from two important rare diseases initiatives, the literature shows that there is large variation in the frequency of rare diseases. Finally, revenue data were obtained from the company’s financial reports, which did not provide audited financial statements, meaning it was not possible to verify the figures, and revenue data in Brazil were limited to public purchases.

## Conclusions

Our findings show that a strategy focused on obtaining orphan drug designation, expanding therapeutic indications and the geographic range of marketing approvals, extending monopoly periods, and prioritizing public procurement niches has enhanced Alexion’s revenues and helped the company achieve leadership in a highly specific market, significantly boosting profitability.

The single-case study approach adopted by this investigation allowed an in-depth examination of these dynamics. Alexion’s granted and pending patents mean that the company has a monopoly on eculizumab that extends to an average of 35 years, keeping competition at bay in major markets. After eculizumab was first designated as an orphan drug by the FDA and EMA in 2003, Alexion obtained a further eight designations in these and other regulatory agencies up to 2014, accompanied by the tax benefits and exclusivity rights provided under the specific legislation of each jurisdiction. Furthermore, an increase in marketing authorizations was observed. With the expansion of therapeutic indications for other rare diseases, the number of patients who could potentially be treated with the drug has increased. These combined factors were and will continue to be essential for promoting a significant increase in the company’s global eculizumab sales revenues.

The situation in Brazil is different to that in the US, EU, and Japan. Specific incentives are not available for designated orphan drugs in Brazil. In this case, the company’s strategy was to secure exclusive public procurement in response to litigation. Moreover, the delay in marketing authorization prevented the entry of biosimilars into the market, thus eliminating competition.

The development and manufacture of innovative products requires major investment. Rare diseases are an ideal market opportunity for pharmaceutical companies aiming to maximize revenues in a short timeframe while maintaining a lean portfolio.

Were drugs not considered to be essential health goods and a necessary means to decrease disease complications, or even to sustain life, Alexion and eculizumab could portray an interesting financial success story. Alexion has dedicated itself to identifying promising products in a number of countries, acquiring intellectual property rights, applying for orphan designation status and marketing authorization, and ultimately expanding clinical indications for eculizumab, making a considerable and sustained profit. In some countries, such as Brazil, company profits have been boosted by belated marketing authorization and litigation to promote access to medicines, resulting in the mandatory purchase of the drug by the government. The systematic way in which the company pursues these strategies constitutes a successful business model.

## Supporting information

S1 Table Alexion Inc. total revenues per year and product portfolio (in million USD), 2007–2019. (DOCX)

S2 Table Alexion Inc. total revenues with eculizumab sales in Brazil and the world (in million USD), 2007–2019. (DOCX)

S1 Data (XLSX)

## References

[1] Herper M. The World’s Most Expensive Drugs. Forbes, 19 fevereiro 2010. https://www.forbes.com/2010/02/19/expensive-drugs-cost-business-healthcare-rare-diseases.html#27d2e8015e10.

[2] Ovans A. What Is a Business Model? Harvard Business Review. 23 January 2015. https://hbr.org/2015/01/what-is-a-business-model. Accessed 2 May 2020.

[3] Lazonick W, Hopkins M, Jacobson K, Sakinç M E, and Tulum, Ö. US Pharma’s Financialized Business Model. New York: Institute for New Economic Thinking (Working Paper N° 62, July 13, 2017, 27 p.). https://www.ineteconomics.org/uploads/papers/WP_60-Lazonick-et-al-US-Pharma-Business-Model.pdf

[4] Simonov R. Dominant business models in the global pharmaceutical industry. Economics & Education 2019;04(02): 39–34. https://ee.isma.lv/images/issues/EE0402/08_EE0402_Simonov.pdf

[5] Orphanet: About Rare Diseases. 2014. https://www.orpha.net/consor/cgi-bin/Education_AboutRareDiseases.php?lng = EN

[6] Richter T, Nestler-Parr S, Babela R, Khan ZM, Tesoro T, et al. Rare Disease Terminology and Definitions-A Systematic Global Review: Report of the ISPOR Rare Disease Special Interest Group. Value Health. 2015;18(6):906–14. 10.1016/j.jval.2015.05.008 . 26409619

[7] Richter T, Janoudi G, Amegatse W, Nestler-Parr S. Characteristics of drugs for ultra-rare diseases versus drugs for other rare diseases in HTA submissions made to the CADTH CDR. Orphanet J Rare Dis. 2018;13(1):15. 10.1186/s13023-018-0762-1 . 29386040 PMC5793441

[8] Graf von der Schulenburg JM, Frank M. Rare is frequent and frequent is costly: rare diseases as a challenge for health care systems. Eur J Health Econ. 2015;16(2):113–8. 10.1007/s10198-014-0639-8 . 25355295

[9] Aronson JK. Rare diseases and orphan drugs. Br J Clin Pharmacol. 2006;61(3):243–5. 10.1111/j.1365-2125.2006.02617.x . 16487216 PMC1885017

[10] Gammie T, Lu CY, Babar ZU. Access to Orphan Drugs: A Comprehensive Review of Legislations, Regulations and Policies in 35 Countries. PloS One. 2015;10(10):e0140002. 10.1371/journal.pone.0140002 . 26451948 PMC4599885

[11] Minghetti PM, Giudici EM, Montanari L. A proposal to improve the supply of orphan drugs. Pharmacol Res. 2000;42(1):33–7. 10.1006/phrs.1999.0644 . 10860632

[12] EvaluatePharma. EvaluatePharma Orphan Drug Report 2019. 6th Edition. April 2019. https://www.evaluate.com/thought-leadership/pharma/evaluatepharma-orphan-drug-report-2019

[13] United States. Orphan drug act, Public Law No. 97–414, 1983. https://www.fda.gov/media/99546/download

[14] European Union. Regulation (EC) No 141/2000 of the European Parliament and of the Council of 16 December 1999 on orphan medicinal products. https://eur-lex.europa.eu/legal-content/EN/TXT/?uri=celex%3A32000R0141

[15] Nagaraja S, Balamuralidhara V, Jyothi MS, Ragunandhan HV. Orphan Drug Regulation in Japan and Australia. Int. J. Res. Pharm. Sci. 2020, 11(2), 1831–1839. 10.26452/ijrps.v11i2.2088

[16] Kondo H. Regulatory/Scientific Support for Rare Disease Product Development in Japan. Office of International Programs Pharmaceuticals and Medical Devices Agency (PMDA). 3rd IRDiRC Conference in February 2017. https://www.irdirc.org/wp-content/uploads/2017/12/PAR3_TRA_02-Kondo.pdf

[17] Government of Brazil. Agência Nacional de Vigilância Sanitária. RDC n° 205 de 28 de dezembro de 2017. Diário Oficial da União, seção 1 p. 28–114. 29 de dezembro de 2015. https://www.in.gov.br/materia/-/asset_publisher/Kujrw0TZC2Mb/content/id/1486126/do1-2017-12-29-resolucao-rdc-n-205-de-28-de-dezembro-de-2017-1486122

[18] Griggs RC, Batshaw M, Dunkle M, et al. Clinical research for rare disease: opportunities, challenges, and solutions. Mol Genet Metab. 2009;96(1):20–6. 10.1016/j.ymgme.2008.10.003 . 19013090 PMC3134795

[19] Hillmen P, Young NS, Schubert J, Brodsky RA, Socié G, Muus P, et al. The complement inhibitor eculizumab in paroxysmal nocturnal hemoglobinuria. N Engl J Med. 2006;355(12):1233–43. 10.1056/NEJMoa061648 . 16990386

[20] Bell, L. The Wall Street Transcript. https://www.twst.com/bio/leonard-bell-m-d-2/

[21] Fierce Biotech. Stephen Squinto, Alexion. https://www.fiercebiotech.com/special-report/stephen-squinto-alexion

[22] Reis ES, Mastellos DC, Yancopoulou D, Risitano AM, Ricklin D, Lambris JD. Applying complement therapeutics to rare diseases. Clin Immunol. 2015;161(2):225–240. 10.1016/j.clim.2015.08.009 . 26341313 PMC4658209

[23] Ricklin D, Mastellos DC, Reis ES, Lambris JD. The renaissance of complement therapeutics. Nat Rev Nephrol. 2018; 14(1): 26–47. 10.1038/nrneph.2017.156 . 29199277 PMC5805379

[24] Mastellos DC, Ricklin D, Lambris JD. Clinical promise of next-generation complement therapeutics. Nat Rev Drug Discov. 2019; 18(9): 707–729. 10.1038/s41573-019-0031-6 . 31324874 PMC7340853

[25] Jayasundara K, Hollis A, Krahn M, Mamdani M, Hoch J, Grootendorst P. Estimating the clinical cost of drug development for orphan versus non-orphan drugs. Orphanet J Rare Dis. 2019;14(1):12. 10.1186/s13023-018-0990-4 . 30630499 PMC6327525

[26] Luzzatto L, Hyry HI, Schieppati A, Costa E, Simoens S, Schaefer F, et al. Outrageous prices of orphan drugs: a call for collaboration. Lancet. 2018;392(10149):791–794. 10.1016/S0140-6736(18)31069-9 . 30037734

[27] Berdud M, Drummond M, Towse A. Establishing a Reasonable Price for an Orphan Drug. OHE Research Paper 18/05, 2018. London: Office of Health Economics. https://www.ohe.org/publications/establishing-reasonable-price-orphan-drug 10.1186/s12962-020-00223-x PMC7472708 32908456

[28] America’s Health Insurance Plans. High-priced drugs: estimates of annual per-patient expenditures for 150 specialty medications. AHIP, 2016. https://www.ahip.org/wp-content/uploads/2016/04/HighPriceDrugsReport.pdf

[29] Crowe S, Cresswell K, Robertson A, Huby G, Avery A, Sheikh A. The case study approach. BMC Med Res Methodol. 2011;11:100. 10.1186/1471-2288-11-100 . 21707982 PMC3141799

[30] Integrity Database. Patents. https://integrity-clarivate.ez83.periodicos.capes.gov.br/integrity/xmlxsl/pk_qcksrch.show_records?sessionID=1&history=&query=alexion&abbreviation=PAT&language=en

[31] United States Patent and Trademark Office (USPTO). https://www.uspto.gov/patents-application-process/search-patents 10.1038/73820 10700156

[32] European Patent Office. Espacenet. Patent Search. https://worldwide.espacenet.com/patent/

[33] Japan Patent Office. The Japan Platform for Patent Information (J-PlatPat). https://www.j-platpat.inpit.go.jp/

[34] Instituto Nacional de Propriedade Intelectual. Consulta a Base de Dados do INPI. http://www.inpi.gov.br/

[35] Google Patents. https://patents.google.com/

[36] US Food and Drug Administration. Orphan Drug Designations and Approvals. https://www.accessdata.fda.gov/scripts/opdlisting/oopd/index.cfm

[37] European Commission. Union Register of medicinal products. https://ec.europa.eu/health/documents/community-register/html/index_en.htm

[38] Japan. Pharmaceuticals and Medical Devices Agency. Orphan Drugs WA. https://www.pmda.go.jp/english/rs-sb-std/rs/0008.html

[39] US Food and Drug Administration. Drugs@FDA: FDA-Approved Drugs. https://www.accessdata.fda.gov/scripts/cder/daf/index.cfm?event=BasicSearch.process

[40] European Medicines Agency. Medicines. https://www.ema.europa.eu/en/medicines

[41] Japan. Pharmaceuticals and Medical Devices Agency (PMDA). http://www.pmda.go.jp/english/review-services/reviews/approved-information/drugs/0001.html#select19

[42] Brasil. Agência Nacional de Vigilância Sanitária–ANVISA. Consulta a Produtos Registrados. Consulta a Medicamentos e Hemoderivados. http://portal.anvisa.gov.br/medicamentos/consultas

[43] Orphanet. https://www.orpha.net/consor/cgi-bin/Disease_Search.php?lng=PT

[44] National Organization for Rare Diseases (NORD). https://rarediseases.org/for-patients-and-families/information-resources/rare-disease-information/

[45] Alexion Pharmaceuticals. Our History. http://alexion.com/about-alexion-pharmaceuticals/history

[46] MarketScreener Homepage. Equities. Nasdaq. Alexion Pharmaceuticals Company—Shareholders, managers and business summary. https://www.marketscreener.com/ALEXION-PHARMACEUTICALS-8334/company/

[47] Cohan, P. S. Disciplined Growth Strategies: Insights from the growth trajectories of successful and unsuccessful companies. Apress, 2017, p. 194.

[48] Herper M. How A $440,000 Drug Is Turning Alexion Into Biotech’s New Innovation Powerhouse. Forbes, Sep 5, 2012. https://www.forbes.com/sites/matthewherper/2012/09/05/how-a-440000-drug-is-turning-alexion-into-biotechs-new-innovation-powerhouse/#2771

[49] Andersson T, Gleadle P, Haslama C, Tsitsianisa N. Bio-pharma: A financialized business model. Critical Perspectives on Accounting 2010; 21(7): 631–641. 10.1016/j.cpa.2010.06.006

[50] Alexion Pharmaceuticals Inc. News & Media. Press Release. https://alexionpharmaceuticalsinc.gcs-web.com/news-media/press-releases

[51] Alexion Pharmaceuticals Inc. Financials. Annual Reports. https://alexionpharmaceuticalsinc.gcs-web.com/financial-information/annual-reports

[52] Brasil. Portal de Compras Governo Federal. SIASG. O que é o Sistema Integrado de Administração de Serviços Gerais. https://www.comprasgovernamentais.gov.br/index.php/sisg/siasg

[53] Instituto de Pesquisa Econômica Aplicada. IPEAdata. Taxa de câmbio comercial para venda: real (R$) / dólar americano (US$)–média. http://www.ipeadata.gov.br/ExibeSerie.aspx?serid=31924&module=M&chart=ChartsImage40417902344583176

[54] Government of Brazil. Lei n° 9.279, de 14 de maio de 1996. Regula direitos e obrigações relativos à propriedade industrial http://www.planalto.gov.br/ccivil_03/leis/l9279.htm

[55] Ducimetière C. Second Medical Use Patents -Legal Treatment and Public Health Issues. Research Paper 101. December 2019. South Centre. https://www.southcentre.int/wp-content/uploads/2019/12/RP101_Second-Medical-Use-Patents-Legal-Treatment-and-Public-Health-Issues_EN.pdf

[56] Amin T, Kesselheim AS. Secondary Patenting of Branded Pharmaceuticals: A Case Study of How Patents on Two HIV Drugs Could Be Extended for Decades. Health Aff 2012;31(10):2286–94. 10.1377/hlthaff.2012.0107 . 23048110

[57] Moorkens E, Vulto AG, Huys I. An overview of patents on therapeutic monoclonal antibodies in Europe: are they a hurdle to biosimilar market entry? MAbs. 2020;12(1):1743517. 10.1080/19420862.2020.1743517 . 32306833 PMC7188399

[58] Chow V, Pan J, Chien D, Mytych DT, Hanes V. A randomized, double-blind, single-dose, three-arm, parallel group study to determine pharmacokinetic similarity of ABP 959 and eculizumab (Soliris®) in healthy male subjects. Eur J Haematol. 2020;105(1):66–74. 10.1111/ejh.13411 . 32196749 PMC7384155

[59] Mehr S. Amgen Settles with Alexion on Soliris Biosimilar, Dropping Litigation and Getting a Release Date. Biosimilar Review & Report. June 16 2020. https://biosimilarsrr.com/2020/06/16/amgen-settles-with-alexion-on-soliris-biosimilar-dropping-litigation-and-getting-a-release-date/#:~:text=a%20Release%20Date-,Amgen%20Settles%20With%20Alexion%20on%20Soliris%20Biosimilar%2C%20Dropping,and%20Getting%20a%20Release%20Date&text=As%20reported%20by%20several%20sources,of%20Soliris%C2%AE%20in%202025

[60] Government of Brazil. Supremo Tribunal da Justiça. RECURSO ESPECIAL N° 1.721.711—RJ (2017/0261991-0). 20 de abril de 2018. https://ww2.stj.jus.br/websecstj/cgi/revista/REJ.cgi/ITA?seq=1699525&tipo=0&nreg=201702619910&SeqCgrmaSessao=&CodOrgaoJgdr=&dt=20180420&formato=PDF&salvar=false

[61] Moorkens E, Jonker-Exler C, Huys I, Declerck P, Simoens S, Vulto AG. Overcoming Barriers to the Market Access of Biosimilars in the European Union: The Case of Biosimilar Monoclonal Antibodies. Front Pharmacol. 2016;7:193. 10.3389/fphar.2016.00193 . 27445826 PMC4925708

[62] Dear JW, Lilitkarntakul P, Webb DJ. Are rare diseases still orphans or happily adopted? The challenges of developing and using orphan medicinal products. Br J Clin Pharmacol. 2006; 62(3): 264–271. 10.1111/j.1365-2125.2006.02654.x . 16934041 PMC1885144

[63] Government of Brazil. Agência Nacional de Vigilância Sanitária. Resolução da Diretoria Colegiada–RDC n° 205, de 28 de dezembro de 2017. Estabelece procedimento especial para anuência de ensaios clínicos, certificação de boas práticas de fabricação e registro de novos medicamentos para tratamento, diagnóstico ou prevenção de doenças raras. Diário Oficial da União n° 249, de 29 de dezembro de 2017.

[64] Vickers PJ. Challenges and opportunities in the treatment of rare diseases. Drug Discovery World 2012;14(2):9–14;16.

[65] Wellman-Labadie O, Zhou Y. The US Orphan Drug Act: rare disease research stimulator or commercial opportunity? Health Policy. 2010;95(2–3):216–28. 10.1016/j.healthpol.2009.12.001 20036435

[66] Roos JC, Hyry HI, Cox TM. Orphan drug pricing may warrant a competition law investigation. BMJ. 2010;341:c6471. 10.1136/bmj.c6471 21081598

[67] Sarpatwari A, Beall RF, Abdurrob A, He M, Kesselheim AS. Evaluating The Impact Of The Orphan Drug Act’s Seven-Year Market Exclusivity Period. Health Aff 2018;37(5):732–737. 10.1377/hlthaff.2017.1179 . 29733729

[68] Brabers AE, Moors EH, van Weely S, de Vrueh RL. Does market exclusivity hinder the development of Follow-on Orphan Medicinal Products in Europe? Orphanet J Rare Dis. 2011;6:59. 10.1186/1750-1172-6-59 21892964 PMC3185263

[69] Attwood MM, Rask-Andersen M, Schiöth HB. Orphan Drugs and Their Impact on Pharmaceutical Development. Trends Pharmacol Sci. 2018;39(6):525–535. 10.1016/j.tips.2018.03.003 . 29779531

[70] Meekings KN, Williams CS, Arrowsmith JE. Orphan drug development: an economically viable strategy for biopharma R&D. Drug Discov Today. 2012 7;17(13–14):660–4. 10.1016/j.drudis.2012.02.005 . 22366309

[71] Giannuzzi V, Conte R, Landi A, Ottomano SA, Bonifazi D, Baiardi P, et al. Orphan medicinal products in Europe and United States to cover needs of patients with rare diseases: an increased common effort is to be foreseen. Orphanet J Rare Dis. 2017;12(1):64. 10.1186/s13023-017-0617-1 . 28372595 PMC5376695

[72] EvaluatePharma. Orphan drug report 2020. London: EvaluatePharma; 2020 April. https://www.evaluate.com/orphan-drugs.

[73] Picavet E, Dooms M, Cassiman D, Simoens S. Drugs for rare diseases: influence of orphan designation status on price. Appl Health Econ Health Policy. 2011;9(4):275–9. 10.2165/11590170-000000000-00000 . 21682354

[74] Brooks M. Rare Disease Treatments Make Up Top 10 Most Costly Drugs. 2 May 2017. https://www.medscape.com/viewarticle/879422.

[75] EvaluatePharma. Orphan drug report 2015. London: EvaluatePharma; 2015 Oct. http://info.evaluategroup.com/rs/607-YGS-364/images/EPOD15.pdf

[76] Röth A, Maciejewski J, Nishimura JI, Jain D, Weitz JI. Screening and diagnostic clinical algorithm for paroxysmal nocturnal hemoglobinuria: Expert consensus. Eur J Haematol. 2018;101(1):3–11. 10.1111/ejh.13059 . 29532535

[77] Noris M, Remuzzi G. Atypical hemolytic-uremic syndrome. N Engl J Med. 2009;361(17):1676–87. 10.1056/NEJMra0902814 . 19846853

[78] Barber C. Diagnosis and management of myasthenia gravis. Nurs Stand. 2017;31(43):42–47. 10.7748/ns.2017.e10434 . 28635482

[79] Suh J, Goldstein JM, Nowak RJ. Clinical characteristics of refractory myasthenia gravis patients. Yale J Biol Med. 2013;86:255–60. . 23766745 PMC3670444

[80] Huda S, Whittam D, Bhojak M, Chamberlain J, Noonan DC, Jacob EA, et al. Neuromyelitis optica spectrum disorders. Clin Med (Lond). 2019;19(2):169–176. 10.7861/clinmedicine.19-2-169 . 30872305 PMC6454358

[81] Auvin S, Irwin J, Abi-Aad P, Battersby A. The Problem of Rarity: Estimation of Prevalence in Rare Disease. Value Health. 2018;21(5):501–507. 10.1016/j.jval.2018.03.002 . 29753345

[82] Lazonick W, Shin J-S. Predatory Value Extraction: How the Looting of the Business Corporation Became the US Norm and How Sustainable Prosperity Can be Restored. Oxford: Oxford University Press, 2020, 256 p.

[83] Lazonick W, Tulum Ö, Hopkins M, Sakinç ME, Jacobson K. Financialization of the U.S. Pharmaceutical industry, The Academic-Industry Research Network. December 2, 2019, 15 p. https://www.ineteconomics.org/perspectives/blog/financialization-us-pharma-industry

[84] Alexion. History. November 17 2020. https://alexion.com/our-company/history.

[85] Fierce Pharma. Leonard Bell, Alexion Pharmaceuticals. https://www.fiercepharma.com/special-report/leonard-bell-alexion-pharmaceuticals.

[86] Alexion. Pipeline. October 29 2020. https://alexion.com/our-research/pipeline.

[87] Hughes DA, Poletti-Hughes J. Profitability and Market Value of Orphan Drug Companies: A Retrospective, Propensity-Matched Case-Control Study. PloS One. 2016;11(10):e0164681. 10.1371/journal.pone.0164681 . 27768685 PMC5074462

[88] Lee JW, Sicre de Fontbrune F, Wong Lee Lee L, Pessoa V, Gualandro S, Füreder W, et al. Ravulizumab (ALXN1210) vs eculizumab in adult patients with PNH naive to complement inhibitors: the 301 study. Blood. 2019;133(6):530–539. 10.1182/blood-2018-09-876136 . 30510080 PMC6367644

[89] Kulasekararaj AG, Hill A, Rottinghaus ST, Langemeijer S, Wells R, Gonzalez-Fernandez FA, et al. Ravulizumab (ALXN1210) vs eculizumab in C5-inhibitor-experienced adult patients with PNH: the 302 study. Blood. 2019;133(6):540–549. 10.1182/blood-2018-09-876805 30510079 PMC6368201

[90] Lee JW, Kulasekararaj AG. Ravulizumab for the treatment of paroxysmal nocturnal hemoglobinuria. Expert Opin Biol Ther. 2020;14:1–11. 10.1080/14712598.2020.1725468 . 32011183

[91] Government of Brazil. Ministério da Saúde, Agência Nacional de Vigilância Sanitária. Consulta ao registro do medicamento Soliris. Brasília, DF: ANVISA; 2019. https://consultas.anvisa.gov.br/#/medicamentos/25351199836201512/?substancia=25890.

[92] Government of Brazil. Ministério da Saúde. Portaria n° 77, de 14 de dezembro de 2018. Torna pública a decisão de incorporar o eculizumabe para tratamento de pacientes com hemoglobinúria paroxística noturna (HPN) no âmbito do Sistema Único de Saúde. Diário Oficial União. 17 dez 2018; Seção 1:76. http://www.in.gov.br/web/guest/materia/-/asset_publisher/Kujrw0TZC2Mb/content/id/55443289/do1-2018-12-17-portaria-n-77-de-14-de-dezembro-de-2018-55443067.

[93] Government of Brazil. Ministério da Saúde. Portaria Conjunta SCTIE/SAES/MS n° 18/2019. Protocolo Clínico e Diretrizes Terapêuticas Hemoglobinúria Paroxística Noturna. Diário Oficial União. 22 nov 2019; Seção 1:153. http://conitec.gov.br/images/Relatorios/Portaria/2019/PortariaConjunta_SCTIE_SAES_18_2019.pdf.

[94] Caetano R, Rodrigues PHA, Corrêa MCV, Villardi P, Osorio-de-Castro CGS. The case of eculizumab: litigation and purchases by the Brazilian Ministry of Health. Rev Saude Publica. 2020;54:22. 10.11606/s1518-8787.2020054001693 32130309 PMC7017980

[95] Vieira MF, Chaves GC. The Patent Paradox in Brazil. implications for purchases of medicines by the public health system. March 2018. Rio de Janeiro: Fiocruz. https://accessibsa.org/media/2018/03/The-Patent-Paradox-In-Brazil.pdf

[96] Government of Brasil. Lei n° 9.782, de 26 de janeiro de 1999. Define o Sistema Nacional de Vigilância Sanitária, cria a Agência Nacional de Vigilância Sanitária, e dá outras providências. http://www.planalto.gov.br/ccivil_03/leis/l9782.htm.

