# Supplementary material for: Dynamics of patents, orphan drug designation, licensing, and revenues from drugs for rare diseases: The market expansion of eculizumab
Source: PLoS One. 2021 Mar 5;16(3):e0247853. doi: 10.1371/journal.pone.0247853 (PMC7935269; doi:10.1371/journal.pone.0247853)
Supplement: S1 Table — (DOCX) [file pone.0247853.s001.docx]

**S1 Table. Alexion Inc. Total Revenues per Year and Product Portfolio (in million USD), 2007-2019**

| **Year** | **Total Revenue** | **Eculizumab Revenues** | **Asfotase alfa Revenues** | **Sebelipase alfa Revenues** | **Ravulizumab Revenues** |
| --- | --- | --- | --- | --- | --- |
| 2007 | 66.4 | 66.4 | Unlicensed | Unlicensed | Unlicensed |
| 2008 | 259.1 | 259.1 | Unlicensed | Unlicensed | Unlicensed |
| 2009 | 386.8 | 386.8 | Unlicensed | Unlicensed | Unlicensed |
| 2010 | 541.0 | 541.0 | Unlicensed | Unlicensed | Unlicensed |
| 2011 | 783.4 | 783.4 | Unlicensed | Unlicensed | Unlicensed |
| 2012 | 1,134.00 | 1,134.00 | Unlicensed | Unlicensed | Unlicensed |
| 2013 | 1,551.0 | 1,551.0 | Unlicensed | Unlicensed | Unlicensed |
| 2014 | 2,234.0 | 2,234.0 | Unlicensed | Unlicensed | Unlicensed |
| 2015 | 2,604.0 | 2,590.0 | 12.0 | 0 | Unlicensed |
| 2016 | 3,084.0 | 2,843.0 | 210.0 | 29.0 | Unlicensed |
| 2017 | 3,549.5 | 3,144.1 | 339.8 | 65.6 | Unlicensed |
| 2018 | 4,130.1 | 3,563.0 | 454.2 | 92.0 | 475.1 |
| 2019 | 4,990.0 | 3,946.4 | 475.1 | 112.2 | 338.9 |

Note: Year of First Market Approval: eculizumab – 2007; asfotase alfa – 2015; sebelipase alfa – 2015; ravulizumab – 2018
