# Supplementary material for: Dynamics of patents, orphan drug designation, licensing, and revenues from drugs for rare diseases: The market expansion of eculizumab
Source: PLoS One. 2021 Mar 5;16(3):e0247853. doi: 10.1371/journal.pone.0247853 (PMC7935269; doi:10.1371/journal.pone.0247853)
Supplement: S2 Table — (DOCX) [file pone.0247853.s002.docx]

**S2 Table. Alexion Inc. Total Revenues with Eculizumab Sales in Brazil and the World (in million USD), 2007-2019**

| **Year** | **Total World Revenues (USD)**  **(A)** | **Total Revenues (USD) in Brazil**  **(B)*** | **B/A**  **(%)** |
| --- | --- | --- | --- |
| 2007 | 66.4 | NA** | NA |
| 2008 | 259.1 | NA** | NA |
| 2009 | 386.8 | 4.4 | 1.15 |
| 2010 | 541.0 | 4.2 | 0.77 |
| 2011 | 783.4 | 15.0 | 1.92 |
| 2012 | 1,134.0 | 56.3 | 4.96 |
| 2013 | 1,551.0 | 270.7 | 17.45 |
| 2014 | 2,234.0 | 503.0 | 22.52 |
| 2015 | 2,590.0 | 1,255.8 | 48.49 |
| 2016 | 2,843.0 | 2,180.0 | 76.68 |
| 2017 | 3,144.1 | 852.6 | 27.12 |
| 2018 | 3,563.0 | 1,634.0 | 45.86 |
| 2019 | 3,946.4 | 1,787.2 | 45.29 |

Legend: NA - Non-applicable

Notes: * - Sale exchange rate: Brazilian real (BRL) / American dollar (USD) - average. In: IPEA - http://www.ipeadata.gov.br/ExibeSerie.aspx?serid=31924&module=M&chart=ChartsImage40417902344583176. ** - No purchases in Brazil
